# Inflamação e No-Reflow: Podem Mudar o Jogo?

**DOI:** 10.36660/abc.20240119

**Published:** 2024-06-03

**Authors:** Ana Teresa Timóteo

**Affiliations:** 1 Unidade Local Saúde São José Hospital Santa Marta Lisboa Portugal Serviço de Cardiologia - Hospital Santa Marta - Unidade Local Saúde São José, Lisboa – Portugal; 2 Faculdade de Medicina da NOVA Lisboa Portugal Faculdade de Medicina da NOVA, Lisboa – Portugal

**Keywords:** Fenômeno de não Refluxo, Inflamação, Intervenção Coronária Percutânea

O no-reflow (NR) é uma possível complicação durante a intervenção coronária percutânea (ICP), particularmente no contexto do infarto do miocárdio com elevação do segmento ST (IAMCSST). É de natureza altamente dinâmica, desenvolve-se gradualmente (ao longo de horas) após a restauração do fluxo sanguíneo coronário e persiste durante dias a semanas, dependendo da gravidade, duração e extensão da isquemia miocárdica e da aplicação de medidas terapêuticas com o objetivo de prevenir ou aliviar a lesão de isquemia/reperfusão.^1^ O NR impacta negativamente nos benefícios proporcionados pela terapia de reperfusão e contribui para maus resultados clínicos.^1 , 2^

O principal mecanismo fisiopatológico do NR é a obstrução microvascular que se desenvolve como consequência de isquemia miocárdica, embolização distal e lesão relacionada à reperfusão.^1^ A frequência de NR após ICP primária difere amplamente dependendo da sensibilidade das ferramentas utilizadas para o diagnóstico e do momento do exame. A angiografia coronária é a mais conveniente, mas subestima a verdadeira frequência do NR.^1^ A ressonância magnética cardíaca (RMC) é o método mais sensível no ambiente clínico, fornecendo informações sobre a presença, localização e extensão da obstrução microvascular.^1^ Com a RMC, a obstrução microvascular é diagnosticada em até 95% dos pacientes com IAMCSST e fluxo TIMI restaurado grau 3, e em 57% dos pacientes com IAMCSST dentro de 7 dias após a ICP primária.^1^ Outras técnicas, como resolução do segmento ST e testes de fisiologia coronariana por cateter, são menos sensíveis ou tecnicamente exigentes.^1^

A isquemia promove agregados de plaquetas, neutrófilos e eritrócitos para as células endoteliais, obstruindo a microcirculação e impedindo o fluxo sanguíneo.^1^ Além disso, isso é ainda amplificado pela lesão da microvasculatura relacionada à reperfusão.^1^ Os neutrófilos levados à microcirculação isquêmica após a restauração do sangue promovem mais agregados e obstrução da microcirculação. Após a infiltração e ativação inicial, os neutrófilos e outras células inflamatórias participam da poderosa resposta inflamatória local e sistêmica que se desenvolve em pacientes com infarto agudo do miocárdio. Esses neutrófilos ativados produzem citocinas inflamatórias, radicais de oxigênio, elastase e metaloproteinases, que causam destruição capilar, vazamento vascular (promovendo edema intersticial e compressão da microcirculação) e forte resposta inflamatória.^1^ Portanto, a isquemia/reperfusão está associada a uma forte resposta inflamatória na zona do infarto, predominantemente mediada por neutrófilos, o que contribui para o NR.

Na edição atual dos Arquivos Brasileiros de Cardiologia, Saylik et al.^3^ investigaram a relação entre o índice prognóstico inflamatório (IPI) e a presença de NR em pacientes com IAMCSST, tratados com ICP primária.^3^ Este novo marcador é calculado pela razão neutrófilos/linfócitos (NLR) multiplicada pela razão proteína C reativa/albumina. Avaliaram 1.541 pacientes e o NR esteve presente em 11,5%. Apresentaram maior IPI e esta associação foi não linear. Ademais, apresentou maior capacidade discriminativa, em comparação com outros marcadores inflamatórios, como índice de imunoinflamação sistêmica, RNL e relação proteína C reativa/albumina. Além disso, melhora a discriminação e o benefício clínico líquido quando adicionado a um modelo de regressão de modelo multivariável de linha de base para a detecção de NR, sendo a variável mais proeminente no modelo completo. Os autores desenvolveram um nomograma baseado no IPI, que apresentou boa capacidade de calibração e discriminação por validação interna de *bootstrap* .

Este é um tópico interessante porque é um marcador facilmente obtido, com implicações importantes no resultado. No entanto, a utilidade deste marcador no ambiente clínico é limitada. A ICP primária é uma intervenção emergente e, portanto, não podemos esperar pelos resultados laboratoriais para iniciar o tratamento. Na coorte do estudo, a maioria dos pacientes foi transferida de hospitais sem ICP para hospitais com ICP, com algum atraso que permitiu um resultado laboratorial antes da ICP. No entanto, este pode não ser o cenário mais frequente na maioria dos países, e não é possível esperar pelos resultados. Outro comentário importante é que os autores definiram "tempo porta-balão" como o tempo entre a admissão do paciente no centro de ICP e o momento da insuflação do balão. Não sabemos qual foi o início dos sintomas e o atraso no primeiro contacto médico, bem como o atraso na transferência do doente e isto poderá ter algumas implicações tanto no resultado como na prevalência de NR. Além disso, o período de inclusão começou em 2013. Naquela época, as estratégias de tratamento não eram as mesmas do tratamento contemporâneo - stents farmacológicos e inibidores potentes de P2Y12 são usados em quase todos os pacientes, e inibidores do receptor da glicoproteína 2b/3a são usados apenas residualmente, em situações de resgate. Isto não foi levado em consideração pelos autores e o tratamento tem um impacto importante no resultado. No início da década de 1990, a frequência de NR (avaliada por angiografia coronariana) era de 11,5% em pacientes submetidos a ICP por infarto agudo do miocárdio, e muito menor (2,7%) no início da década de 2010.^4 , 5^ Portanto, não foi avaliado o impacto do IPI em uma coorte com tratamento recomendado pelas diretrizes contemporâneas.

Um comentário final está relacionado às reais implicações clínicas deste marcador. Apesar de décadas de intensa pesquisa e testes de inúmeras estratégias terapêuticas, pouco progresso foi feito na descoberta de uma estratégia de tratamento de eficácia comprovada a ser usada rotineiramente para prevenir NR em pacientes com IAMCSST.^1^ Todos os mecanismos fisiopatológicos de obstrução microvascular e NR têm sido alvo de estratégias preventivas não farmacológicas ou farmacológicas como estratégias únicas ou combinadas. Estratégias como implante de stent direto, dispositivos de proteção contra embolização distal, trombectomia mecânica, inibidores dos receptores da glicoproteína 2b/3a, adenosina, nitrato de sódio, bloqueadores dos canais de cálcio, betabloqueadores, estatinas, fibrinólise intracoronária, bivalirudina, epinefrina intracoronária foram testadas.^1^ No entanto, nenhuma terapia satisfatória foi encontrada para prevenir ou reverter esses fenômenos e melhorar consistentemente o resultado clínico em pacientes com IAMCSST submetidos à reperfusão. Portanto, a prevenção ou o alívio da obstrução microvascular e do NR permanecem metas não alcançadas na terapia do IAMCSST. Embora o presente estudo possa apenas apontar para uma associação entre NR e IPI (porque o desenho do estudo transversal não pode determinar uma relação causal), ele sugere que estratégias direcionadas à inflamação devem ser buscadas para abordar o NR, seja para prevenção ou tratamento.
