# Supplementary material for: Inflamação e No-Reflow: Podem Mudar o Jogo?
Source: Arq Bras Cardiol. 2024 Jun 3;121(5):e20240119. [Article in Portuguese] doi: 10.36660/abc.20240119 (PMC11164438; doi:10.36660/abc.20240119)
Supplement: Translation

# Translation: Inflammation and No-Reflow: Can it be a Game-Changer?

**DOI:** 10.36660/abc.20240119i

**Authors:** Ana Teresa Timóteo

**Affiliations:** 1 Lisboa Portugal Serviço de Cardiologia - Hospital Santa Marta - Unidade Local Saúde São José, Lisboa – Portugal; 2 Lisboa Portugal Faculdade de Medicina da NOVA, Lisboa – Portugal

**Keywords:** No-Reflow Phenomenon, Inflammation, Percutaneous Coronary Intervention

No-reflow (NR) is a possible complication during percutaneous coronary intervention (PCI), particularly in the context of ST-segment elevation myocardial infarction (STEMI). It is highly dynamic in nature, develops gradually (over hours) following coronary blood flow restoration, and persists over days to weeks depending on severity, duration, and extent of myocardial ischemia and application of therapeutic measures aiming to prevent or alleviate ischemia/reperfusion injury.^1^NR impacts negatively on the benefits provided by reperfusion therapy and contributes to poor clinical outcomes.^1 , 2^

The main pathophysiological mechanism of NR is microvascular obstruction developing as a consequence of myocardial ischemia, distal embolization, and reperfusion-related injury.^1^The frequency of NR after primary PCI differs widely depending on the sensitivity of the tools used for diagnosis and the timing of examination. Coronary angiography is the most convenient, but it underestimates the true frequency of NR.^1^Cardiac magnetic resonance (CMR) imaging is the most sensitive method in the clinical setting, providing information on the presence, localization, and extent of microvascular obstruction.^1^With CMR, microvascular obstruction is diagnosed in up to 95% of patients with STEMI and restored TIMI flow grade 3, and in 57% of patients with STEMI within 7 days after primary PCI.^1^Other techniques, such as ST-segment resolution and catheter-based coronary physiology tests are less sensitive or technically demanding.^1^

Ischemia promotes aggregates of platelets, neutrophils, and erythrocytes to endothelial cells, clogging the microcirculation and impeding blood flow.^1^In addition, this is further amplified by reperfusion-related microvasculature injury.^1^Neutrophils brought to ischemic microcirculation upon blood restoration, promote more aggregates and microcirculation obstruction. Following initial infiltration and activation, neutrophils, and other inflammatory cells participate in the powerful local and systemic inflammatory response that develops in patients with acute myocardial infarction. These activated neutrophils produce inflammatory cytokines, oxygen radicals, elastase, and metalloproteinases, which cause capillary destruction, vascular leakage (promoting interstitial edema and microcirculation compression), and a strong inflammatory response.^1^Therefore, ischemia/reperfusion is associated with a strong inflammatory response in the infarct zone, predominantly mediated by neutrophils, which contributes to NR.

In the current issue of *Arquivos Brasileiros de Cardiologia* , Saylik et al.^3^investigated the relationship between inflammatory prognostic index (IPI) and the presence of NR in patients with STEMI, treated with primary PCI.^3^This novel maker is calculated by neutrophil/lymphocyte ratio (NLR) multiplied by the C-reactive protein/albumin ratio. They assessed 1541 patients, and NR was present in 11.5%. They showed higher IPI and this association was non-linear. Moreover, it showed higher discriminative ability, compared to other inflammatory markers, such as systemic immune-inflammation index, NLR, and C-reactive protein/albumin ratio. In addition, it improves discrimination and net-clinical benefit when added to a baseline multivariable model regression model for the detection of NR, being the most prominent variable in the full model. The authors developed a nomogram based on IPI, that showed good calibration and discrimination ability by bootstrap internal validation.

This is an interesting topic because this is an easily obtainable marker, with important implications on outcome. However, the utility of this marker in the clinical setting is limited. Primary PCI is an emergent intervention, and therefore, we cannot wait for laboratory results to start the treatment. In the study cohort, most patients were transferred from non-PCI hospitals to PCI hospitals, with some time delay that allowed a laboratory result before PCI, However, this might not be the most frequent scenario in most countries, and it is not possible to wait for the results. Another important comment is that the authors defined "door-to-balloon time" as the time between the patient's admission to the PCI center and the time of balloon inflation. We do not know what was the symptoms onset to first medical contact delay, as well as the patient transfer delay and this might have some implications both on the outcome and in the prevalence of NR. Moreover, the inclusion period started in 2013. At that time, treatment strategies were not the same as contemporary treatment - drug-eluting stents and potent P2Y12 inhibitors are used in almost every patient, and glycoprotein 2b/3a receptor inhibitor are used only residually, in bail-out situations. This was not taken into account by the authors and treatment has an important impact on outcome. In the early 1990s, the frequency of NR (assessed by coronary angiography) was 11.5% in patients undergoing PCI for acute myocardial infarction, and much lower (2.7%) in the early 2010s.^4 , 5^Therefore, it was not assessed the impact of the IPI in a cohort with contemporaneous guideline-recommended treatment.

A final comment is related to the real clinical implications of this marker. Despite decades of intensive research and testing of numerous therapeutic strategies, little progress has been made in finding a treatment strategy of proven efficacy to be routinely used to prevent NR in patients with STEMI.^1^All pathophysiological mechanisms of microvascular obstruction and NR have been targeted by nonpharmacological or pharmacological preventive strategies as single or combined strategies. Strategies like direct stenting, distal embolization protective devices, mechanical thrombectomy, glycoprotein 2b/3a receptor inhibitors, adenosine, sodium nitrate, calcium channel blockers, beta-blockers, statins, intracoronary fibrinolysis, bivalirudine, intracoronary epinephrine have been tested.^1^However, no satisfactory therapy has been found to prevent or reverse these phenomena and consistently improve the clinical outcome in patients with STEMI undergoing reperfusion. Therefore, the prevention or alleviation of microvascular obstruction and NR remain unmet goals in the therapy of STEMI. Although the current study can only point to an association between NR and IPI (because the cross-sectional study design cannot determine a causal relationship) it suggests that strategies targeting inflammation should be sought to address NR, either for prevention or treatment.
